# An analysis of the dynamic spatial spread of COVID-19 across South Korea

**DOI:** 10.1038/s41598-022-13301-2

**Published:** 2022-06-07

**Authors:** Dayun Kang, Jungsoon Choi, Yeonju Kim, Donghyok Kwon

**Affiliations:** 1 Department of Applied Statistics, Hanyang University, Seoul, Republic of Korea; 2 Department of Mathematics, Hanyang University, Seoul, Republic of Korea; 3 Research Institute for Natural Sciences, Hanyang University, Seoul, Republic of Korea; 4 Division of Public Health Emergency Response Research, Korea Disease Control and Prevention Agency, Cheongju, Republic of Korea

**Keywords:** Public health, Diseases, Infectious diseases

## Abstract

The first case of coronavirus disease 2019 (COVID-19) in South Korea was confirmed on January 20, 2020, approximately three weeks after the report of the first COVID-19 case in Wuhan, China. By September 15, 2021, the number of cases in South Korea had increased to 277,989. Thus, it is important to better understand geographical transmission and design effective local-level pandemic plans across the country over the long term. We conducted a spatiotemporal analysis of weekly COVID-19 cases in South Korea from February 1, 2020, to May 30, 2021, in each administrative region. For the spatial domain, we first covered the entire country and then focused on metropolitan areas, including Seoul, Gyeonggi-do, and Incheon. Moran’s I and spatial scan statistics were used for spatial analysis. The temporal variation and dynamics of COVID-19 cases were investigated with various statistical visualization methods. We found time-varying clusters of COVID-19 in South Korea using a range of statistical methods. In the early stage, the spatial hotspots were focused in Daegu and Gyeongsangbuk-do. Then, metropolitan areas were detected as hotspots in December 2020. In our study, we conducted a time-varying spatial analysis of COVID-19 across the entirety of South Korea over a long-term period and found a powerful approach to demonstrating the current dynamics of spatial clustering and understanding the dynamic effects of policies on COVID-19 across South Korea. Additionally, the proposed spatiotemporal methods are very useful for understanding the spatial dynamics of COVID-19 in South Korea.

## Introduction

After the coronavirus disease 2019 (COVID-19) outbreak in Wuhan, China, in late December 2019^1^, the number of cases of COVID-19 in most countries, including China, dramatically increased. The World Health Organization (WHO) reported 95,324 confirmed cases with 3281 deaths globally by March 5, 2020, and declared COVID-19 a pandemic on March 11, 2020^2^.

South Korea was one of the first countries to announce and respond to a COVID-19 case. On January 20, 2020, the first confirmed case was reported, and 30 confirmed cases were reported by February 17, 2020. After the 31st patient was identified on February 18, 2020, attending Shincheonjii church services in Daegu (located in the country’s southeast region), the number of newly confirmed patients dramatically increased to approximately 750 by February 24, 2020 (6 days). As of September 15, 2021, 277,989 confirmed cases and 2380 deaths of COVID-19 were reported in South Korea (https://kdca.go.kr).

The Korean government implemented various strategies, including rapid diagnostic testing, social distancing, and wearing face masks nationwide, to control and reduce the spread of COVID-19. In addition, different COVID-19 control policies at the local administrative level have been implemented based on the volume of cases. It is possible to visualize the dynamics of the disease from the results of spatial and temporal analysis of COVID-19 confirmed cases at the local administrative level, which may help us understand epidemics of the newly emergent infectious disease.

In many countries, various spatial or spatiotemporal analyses of COVID-19 have been performed to understand the characteristics of epidemics and evaluate public health policies. In China, the spatial spread of COVID-19 cases at the early stage was investigated^3,4^, and the spatiotemporal characteristics of COVID-19 transmission in 31 provincial-level regions and 337 prefecture-level cities were examined^5^. In the United States, the dynamic spatial spread of COVID-19 at the state level using metric geometry was analyzed^6^, and spatiotemporal clusters of county-level daily COVID-19 cases were detected from January 22nd to March 27th, 2020^7^. Additionally, the patterns of COVID-19 cases in rural and urban areas were compared, showing different temporal and spatial distributions^8,9^. In the UK, the spatial distribution of COVID-19 cases was explored and regional outbreaks were detected^10^. The spatiotemporal distribution of COVID-19 infection using unaggregated data was explored^11^. Daily COVID-19 cases and deaths in Brazil were used to explore their spatial patterns^12^. The spatiotemporal distribution of local-level COVID-19 cases in Italy was modeled and a significant impact of strict control policies on the spread was found^13^.

The spatiotemporal dynamics of COVID-19 may be influenced by various local confounding factors^14^. For example, any intervention effects, such as social distancing or vaccination rates, may be related to COVID-19 spread^15–17^. Several studies have investigated the effects of air pollution, climate, and weather-related factors, such as temperature, wind, and humidity, on COVID-19 spread^18–23^. In addition, the spatial associations between COVID-19 and population mobility and demographic characteristics have been discussed^14,24^.

Several studies have examined spatially dependent effects or detected spatial clusters using Moran’s I statistics and spatial scan statistics in China^3–5^ and Iran^25,26^. Additionally, the spatial association between COVID-19 and the government response in South Korea at the early stage, from January 20 to May 2020 was assessed^27^. Following the COVID-19 outbreak in 2020, the spatial diffusion and patterns of COVID-19 have varied dynamically, depending mainly on the control policy, human mobility, and epidemic mechanism. When the outbreaks or the size of the high-risk spatial clusters increased, the government might have implemented a stronger social distancing policy at the national level or in high-risk areas to control COVID-19 transmission and reduce the spread of the virus. Thus, it is important to understand and investigate the dynamic spatial patterns of COVID-19 over a longer period.

In this study, we conducted a spatiotemporal analysis of confirmed COVID-19 cases across South Korea from February 18, 2020, to May 31, 2021, to investigate the spatial and temporal variations in COVID-19 and identify the temporally varying spatial cluster patterns of COVID-19 in South Korea.

## Data and methods

### Data sources

To investigate the spatial dynamics of COVID-19 cases across South Korea, the district-level (called si/gun/gu) number of daily or weekly COVID-19 cases was needed. However, the district-level COVID-19 dataset across South Korea was not publicly available, and there were no real figures. Thus, we used the official daily confirmed COVID-19 cases by district obtained by the Korea Disease Control and Prevention Agency. In this study, we analyzed district-level weekly cases from February 18, 2020, to May 31, 2021, in 250 districts across South Korea. The daily statistics of COVID-19 cases in South Korea include information on whether the case was infected outside or inside the country. Because we focused on local transmission within the community, cases from foreign countries were excluded from the study. All methods were performed in accordance with relevant guidelines and regulations as reviewed and approved by the Institutional Review Boards of Hanyang University Seoul Hospital (HYU-2019-04-021).

### Research methods

#### Global Moran’s I

Moran’s I statistic measures spatial autocorrelation^28^ and is defined as follows:

$$I=\frac{n\sum_{i,j}{W}_{ij}({X}_{i}-\overline{X })({X}_{j}-\overline{X })}{\sum_{i\ne j}{W}_{ij}{\sum }_{i}{\left({X}_{i}-\overline{X }\right)}^{2}},$$

where $i$ and $j$ are the region indices and the element ${W}_{ij}$ is the adjacency between areas $i$ and $j$. We set ${W}_{ij}$ to 1 if areas $i$ and $j$ shared a border and 0 if otherwise. The variables ${X}_{i}$ and ${X}_{j}$ denote the number of new confirmed cases in areas $i$ and $j$, respectively, and $\overline{X }$ indicates the average number of new confirmed cases in the area. A value of 0 implies complete spatial randomness in the data. If Moran’s $I$ value is larger than 0, it indicates the clustering of similar values, whereas a negative value indicates the clustering of distinct values. A large absolute value of Moran’s $I$ implies a strong spatial autocorrelation. The mathematical formula of the statistic is similar to the Pearson correlation coefficient, but Moran’s $I$ is not bounded in $[-\mathrm{1,1}]$. Some alternative versions of Moran’s I were proposed to explain heterogeneous populations or consider various weight functions^29–31^.

In this study, we focused mainly on the spatial autocorrelation among COVID-19 cases, not adjusting the population sizes. In the weight function formula, the definition of the geographic distance for our irregular district-level data is not clear. Thus, the original Moran’s I with the adjacent weight function was considered in the analysis.

### Spatial scan statistic

The spatial scan statistic is a typical statistic for spatial cluster detection^32^. The scan statistic ${\lambda }_{z}$ is defined using the likelihood function as follows:

$${\lambda }_{z}=\frac{\underset{z\in Z, {H}_{a}}{max}L(\theta |z)}{\underset{z\in Z, {H}_{0}}{max}L(\theta |z) }=\underset{z\in Z}{\mathit{max}}LR(z),$$

where $z$ and $Z$ denote a scanning window in the spatial domain and the collection of all scanning windows, respectively. Here, $L(\theta |z)$ is the likelihood function. The null hypothesis ${H}_{0}$ is that a spatial cluster does not exist in the spatial domain. Alternatively, hypothesis ${H}_{a}$ is that a certain cluster does exist in the spatial domain. The size of the scanning windows can vary and usually does not exceed 50% of the study domain^33^. Various probability distributions can be assumed appropriately for the data. Our COVID-19 data have excess zeros at some weeks. Thus, this study assumed a zero-inflated Poisson distribution if the number of areas with zero cases exceeded 30% of the total and the Poisson distribution if otherwise. The maximum size of the scanning window was set to 20%. We defined the scanning window $z$ with the maximum ${\lambda }_{z}$ as the most likely cluster. Monte Carlo hypothesis testing is widely used to obtain the p value of the most likely cluster. We simulated 999 random datasets for Monte Carlo testing. Additionally, we chose the most likely cluster as the final spatial cluster only if the number of cases for each area was above the 90th percentile.

For analysis, we used R statistical software (version 3.6.3; https://www.r-project.org/) using the ‘SpatialEpi’^34^ and ‘scanstatistics’^35^ packages for the spatial scan statistic. We used the ‘ape’ package for Moran’s $I$ statistic^36^. In addition, all the figures were created using R software.

### Ethical approval

No human or animal samples were included in the research presented in this article; therefore, ethical approval was not necessary for this research.

## Results

### Weekly incidence of COVID-19 cases

**Figure 1:**
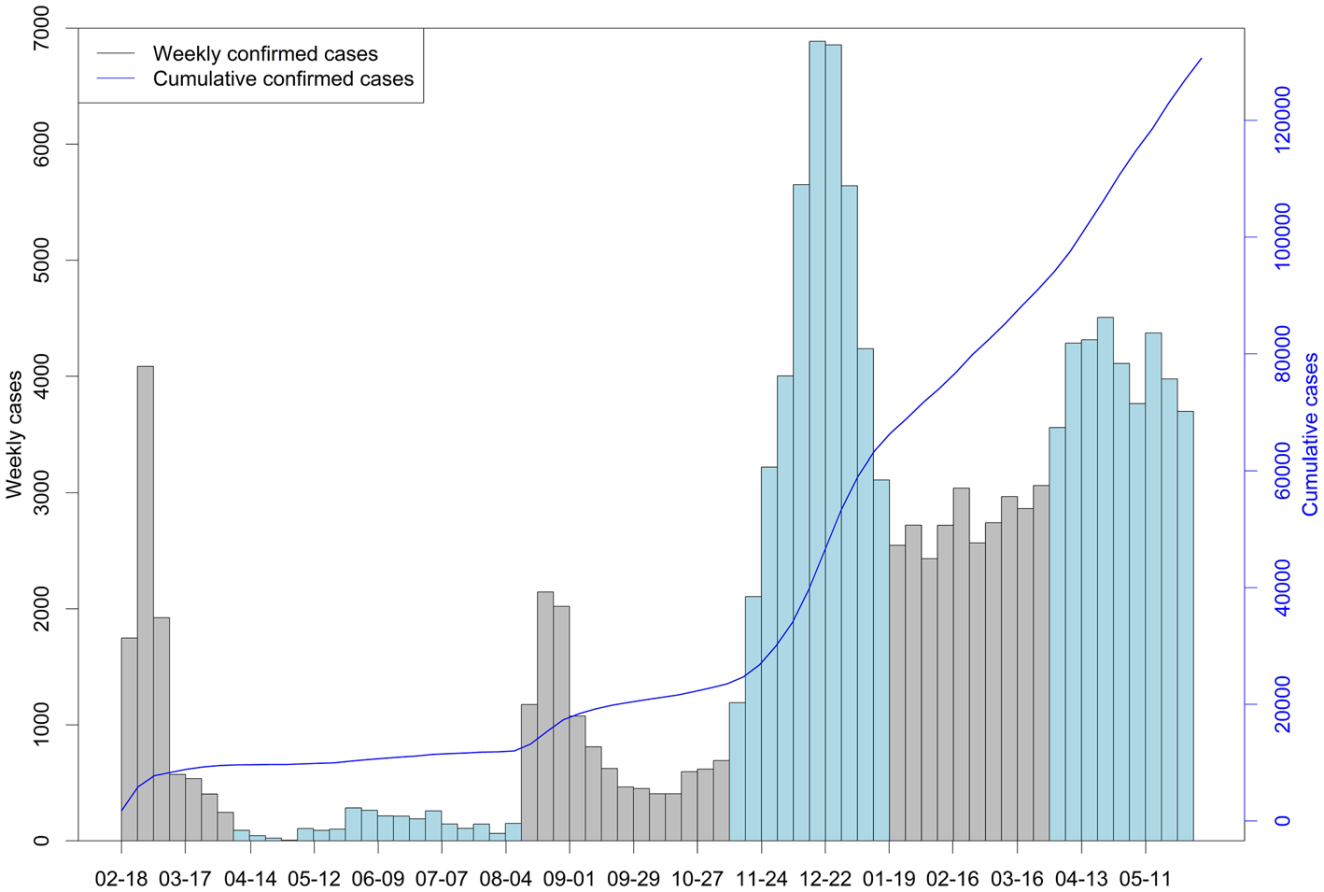
Time series plot for weekly confirmed cases and cumulative confirmed cases of COVID-19 in South Korea from February 18, 2020, to May 11, 2021 (The bar colors distinguish the six temporal periods based on Table 1).

Figure 1 presents the time series plots of the newly confirmed cases and the cumulative confirmed cases every week. Bars indicate the weekly new cases with the left axis, and the blue line indicates the cumulative cases with the right axis. Within the temporal domain of the study, from February 18, 2020, to May 31, 2021, the highest count of new cases was 6887 between December 15 and December 21, 2020 (inclusive). A total of 132,060 patients were diagnosed with COVID-19 during the study period. To understand and compare temporal patterns of the weekly number of cases, we divided the dataset into six periods based on the number of cases. If the number of cases at each week was greater/less than the mean plus/minus standard deviation of the number of cases for the previous three weeks and the period length was greater than 4 weeks, then the new period was determined. Table 1 provides summary statistics for each period. The number of new cases was the highest from November 10, 2020, to January 18, 2021 (weekly mean of 4290 cases) and the lowest from April 7 to August 10, 2021 (weekly mean of 138 cases).

**Table 1:** Summary statistics for the number of weekly cases in six periods in South Korea.

Period	Mean	SD	Min	Q1	Q2	Q3	Max
Feb 18–Apr 6, 2020	1359	1375	245	470	573	1836	4085
Apr 7–Aug 10, 2020	138	83	6	90	125	207	284
Aug 11–Nov 9, 2020	883	583	406	464	624	1076	2145
Nov 10, 2020–Jan 18, 2021	4290	1943	1191	3138	4120	5649	6887
Jan 19–Mar 29, 2021	2766	214	2433	2606	2732	2941	3062
Mar 30–May 31, 2021	4065	334	3558	3764	4110	4314	4507

*Min* Minimum, *SD* standard deviation, *Q1* first quartile, *Q2* second quartile, *Q3* third quartile, *Max* maximum.

After February 18, 2020, the number of confirmed cases increased dramatically until the beginning of March 2020. During this period, mass transmission occurred in Daegu and Gyeongsangbuk-do. From February 18 to March 9, 2020, a total of 7021 patients were diagnosed with COVID-19 in Daegu and Gyeongsangbuk-do, which was 90% of the total number of COVID-19 patients in South Korea in this period. Later, the number of new infections has greatly increased again since November 2020, mainly in metropolitan areas, including Seoul, Gyeonggi, and Incheon. From December 2020 to May 2021, a total of 68,952 cases were reported from Seoul, Gyeonggi, and Incheon, which is 68% of the cases in the entire country in the period. The weekly cases have never been less than 3000 cases since April 2021.

**Figure 2:**
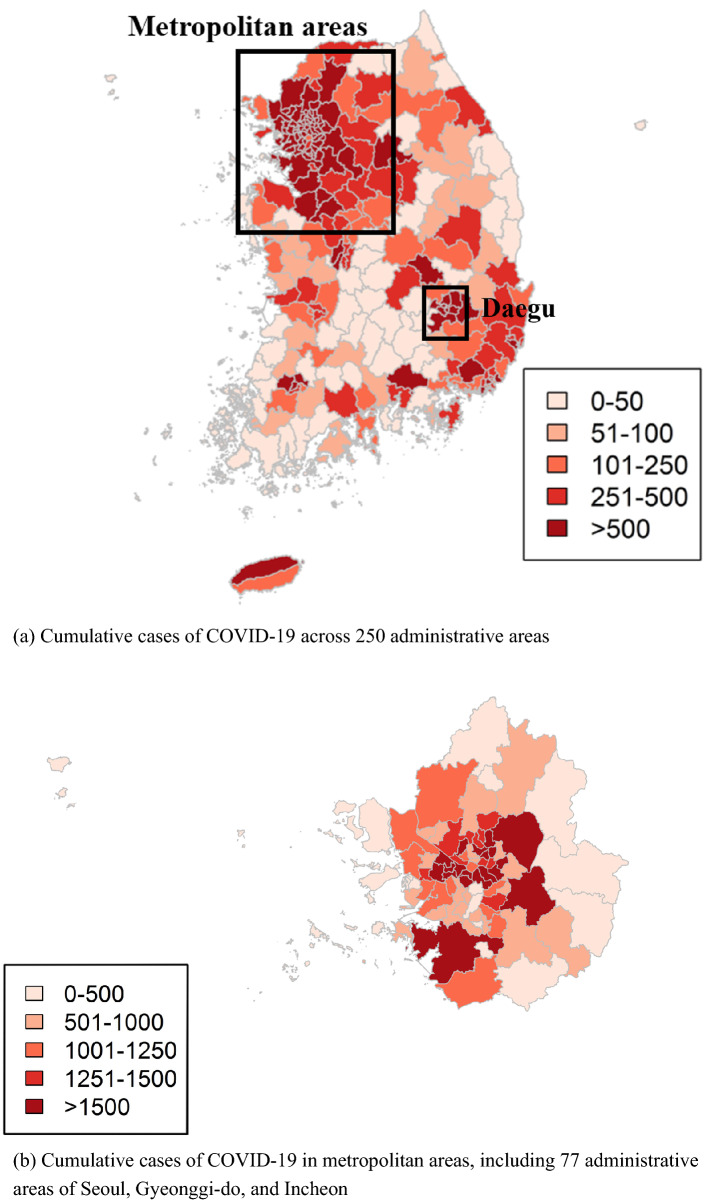
Map of the cumulative confirmed cases of COVID-19 in South Korea from February 18, 2020, to May 31, 2021.

To investigate the geographical distribution of the number of cases, we produced a map of the cumulative cases for 250 administrative areas of South Korea (Fig. 2a) and 77 administrative areas of three metropolitan cities of Seoul, Gyeonggi, and Incheon (Fig. 2b). The cases were the highest around metropolitan areas and Daegu. Moreover, a strong spatial dependency was uncovered, and most of the areas in Seoul had more than 1000 cases.

### Spatiotemporal analysis over the entire area

**Figure 3:**
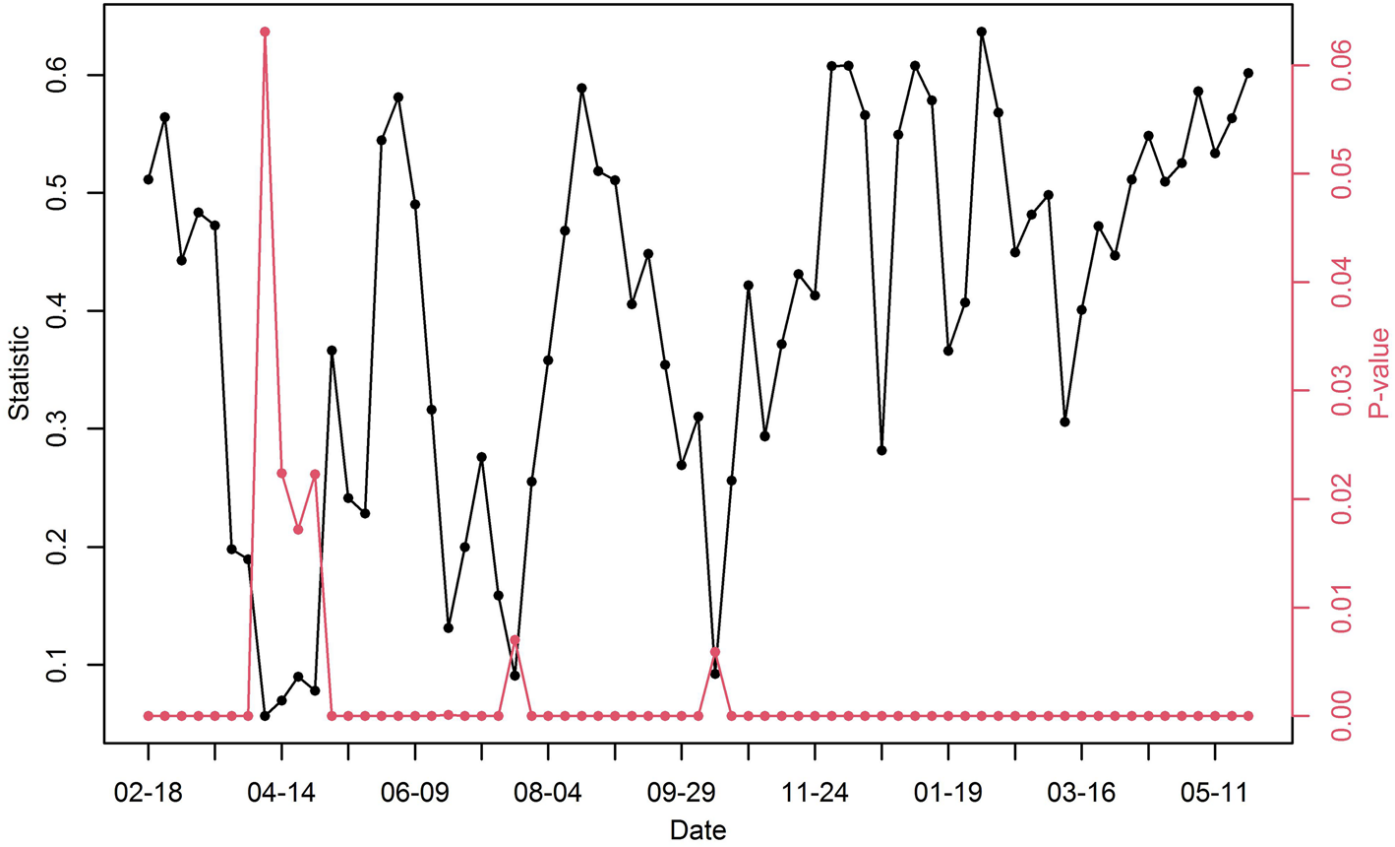
Time series plot for global Moran’s $I$ statistic (black line) and p value (red line) of COVID-19 cases for each week in South Korea from February 18, 2020, to May 11, 2021.

We calculated the global Moran’s $I$ statistic for each week over the entire area to check the spatial association in the number of confirmed cases. In Fig. 3, the black and red lines indicate the statistic and its p value, respectively. The p values of Moran’s I were less than 0.0001 at 61 weeks (approximately 91% of the time domain), showing highly significant spatial autocorrelation. Additionally, p values at 5 weeks were between 0.005 and 0.025, providing medium significant spatial autocorrelation. When the number of new cases dramatically increased, the statistics also tended to increase, such as in August and November 2020. This implies that the coronavirus spread spatially when the number of new infections increased. In particular, in 2021, the statistic tends to increase from March 2021.

**Figure 4:**
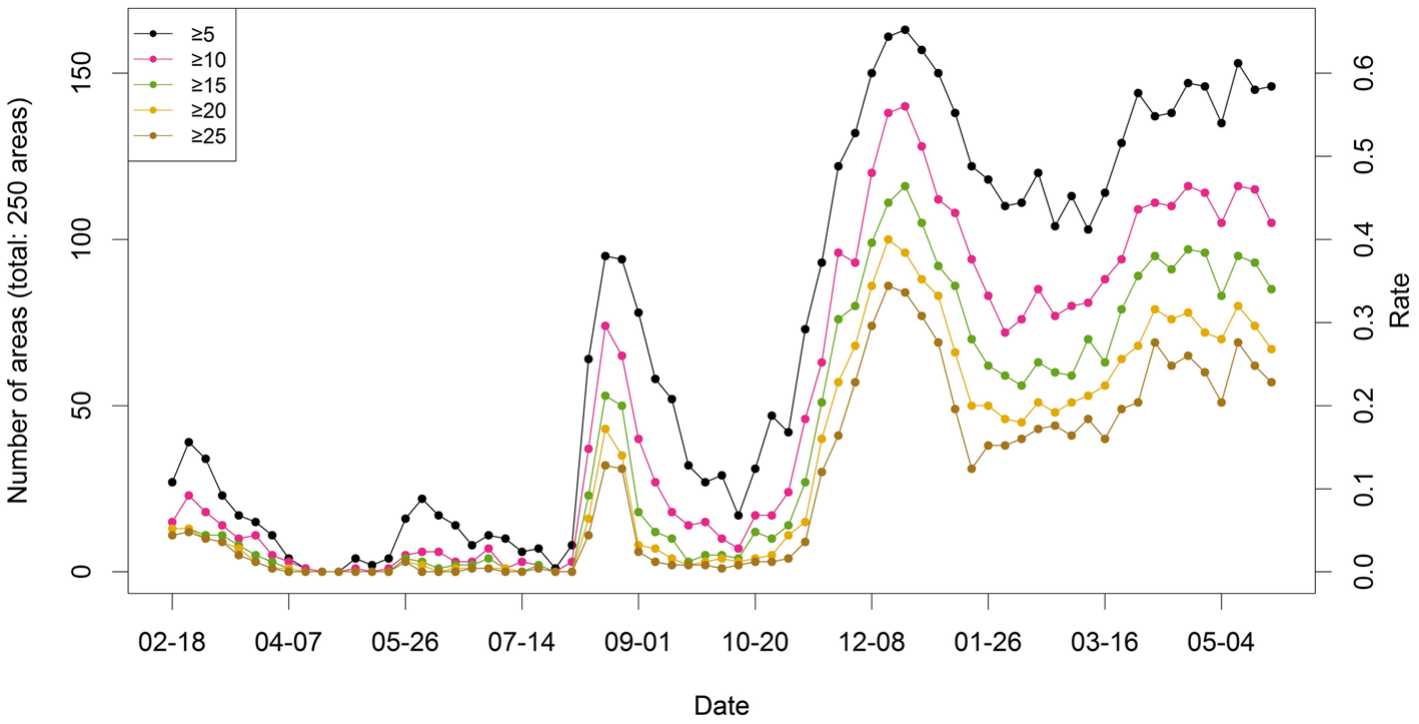
Time series plot for the number of areas with COVID-19 cases over a threshold in South Korea from February 18, 2020, to May 11, 2021.

In addition to Moran’s $I$, we calculated the number of areas with a higher number of cases than a threshold (5, 10, 15, 20, and 25 cases) for each week to investigate the spatial diffusion, as shown in Fig. 4. The larger the number of areas is, the more active the spatial spread. The left side of the $y$-axis denotes the number of areas, and the right side indicates the number of areas divided by the total number of areas (250 areas). All five lines show a similar temporal tendency to Moran’s $I$ statistics in Fig. 3. This pattern indicates that the virus spread actively during the peak seasons in South Korea. For example, before August 2020, less than 20% of 250 areas had more than five cases. In contrast, after November 2020, over 50% of 250 areas had more than five cases.

**Figure 5:**
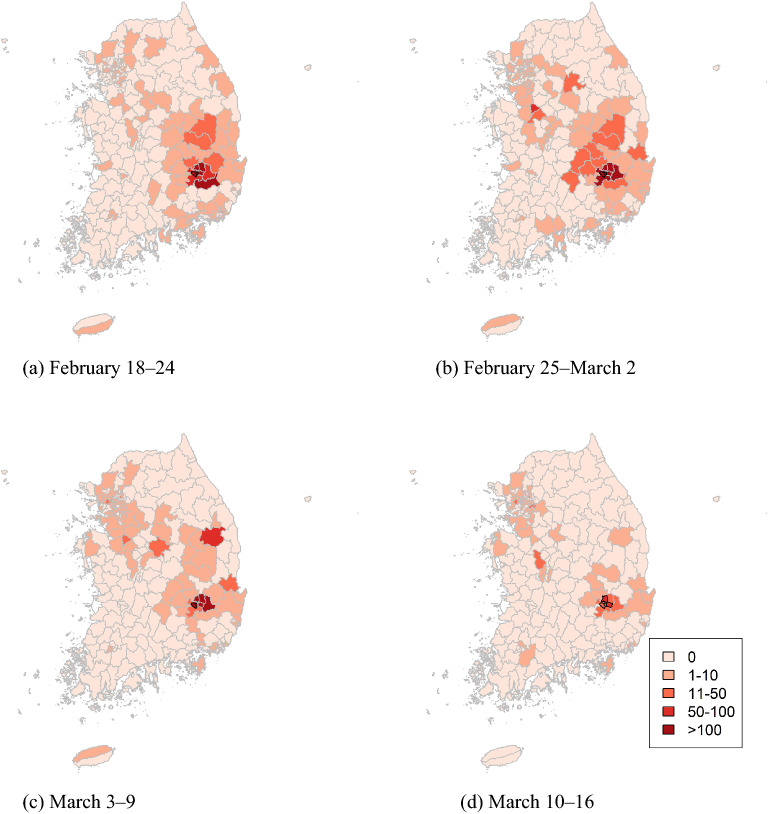
Cluster maps of COVID-19 in South Korea from February 18 to March 10, 2020.

To detect the spatial cluster with elevated risks, we used the spatial scan statistic for two peak seasons: the first from February 18, 2020, to mid-March 2020, and the second from December 1 to December 28, 2020. During the first peak season, the areas in Daegu were detected as clusters (Fig. 5, Table 2): the areas with black borderlines in Fig. 5 represent the clusters. During this period, the number of new infections mainly developed in Daegu and Gyeongsangbuk-do.

**Table 2:** Cluster information of COVID-19 in South Korea from February 18 to March 10, 2020.

Week	Areas	Observed cases	Expected cases	Relative risk	p value
February 18–24	Daegu Nam-gu Daegu Dalseo-gu	686	13.98	2.67	0.001
February 25–March 2	Daegu Nam-gu Daegu Dalseo-gu	1633	32.68	1.39	0.001
March 3–9	Daegu Dalseo-gu	369	7.69	2.47	0.001
March 10–16	Daegu Nam-gu Daegu Dalseo-gu Daegu Seo-gu Daegu Buk-gu Daegu Suseong-gu	225	11.46	2.2	0.001

**Figure 6:**
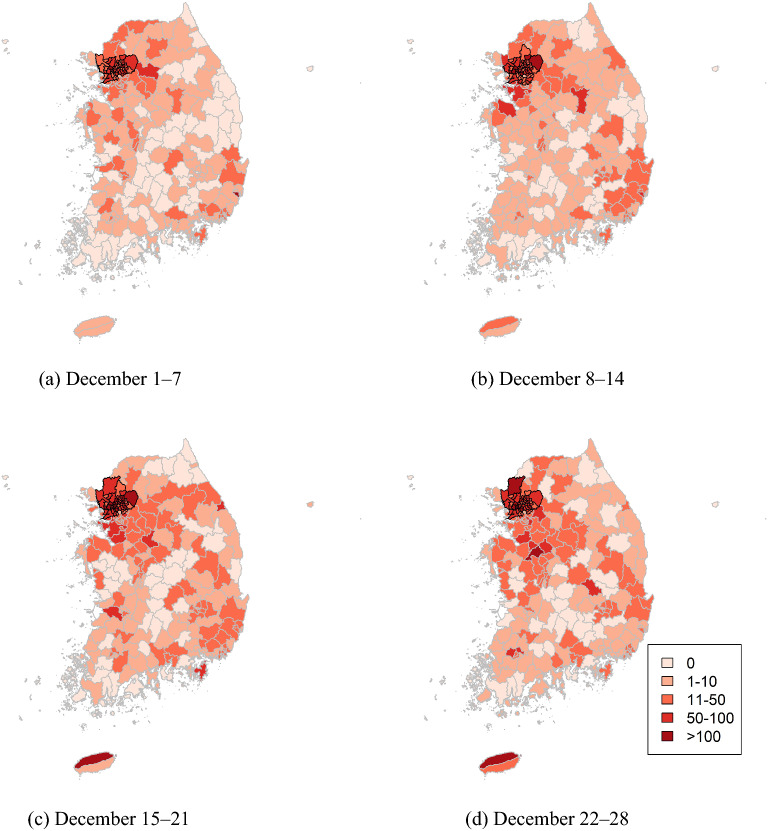
Cluster maps of COVID-19 in South Korea in December 2020.

Unlike the first peak, all the clusters were in metropolitan areas in December 2020 (Fig. 6, Table 3). Most of them were in Seoul, and some were in Gyeonggi and Incheon. As shown in the maps, the number of cases was focused in metropolitan areas at the beginning of December, and the number increased in other areas as the coronavirus spread geographically.

**Table 3:** Cluster information of COVID-19 in South Korea in December 2020.

Week	Areas	Observed cases	Expected cases	Relative risk	p value
December 1–7	Seoul Jongno-gu and 23 other areas Gyeonggi Bucheon-si and 12 other areas Incheon Bupyoung-gu and 2 other areas	2381	640.48	3.36	0.001
December 8–14	Seoul Jongno-gu and 24 other areas Gyeonggi Bucheon-si and 18 other areas Incheon Bupyoung-gu and 3 other areas	3514	1085.18	3.13	0.001
December 15–21	Seoul Jongno-gu and 24 other areas Gyeonggi Bucheon-si and 15 other areas Incheon Bupyoung-gu and 2 other areas	4007	1212.11	3.18	0.001
December 22–28	Seoul Jongno-gu and 24 other areas Gyeonggi Bucheon-si and 14 other areas Incheon Bupyoung-gu and 2 other areas	3893	1179.23	3.12	0.001

### Spatiotemporal analysis over metropolitan areas

**Figure 7:**
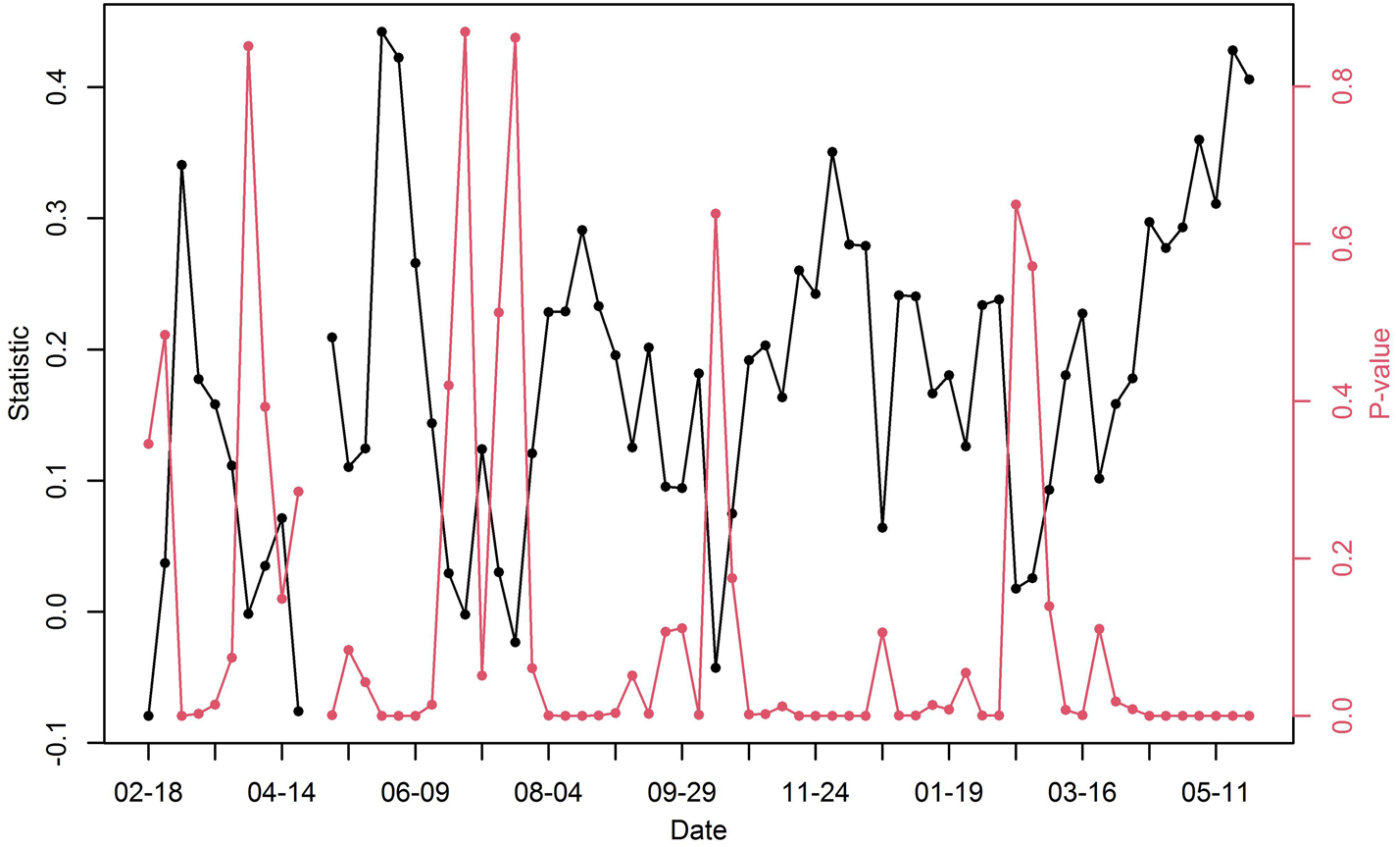
Time series plot for Moran’s $I$ statistic and p value for COVID-19 cases in each week in metropolitan areas in South Korea from February 18, 2020, to May 11, 2021 (no calculation of Moran’s I on the week, April 28 to May 4, 2020, due to the lack of data information).

**Figure 8:**
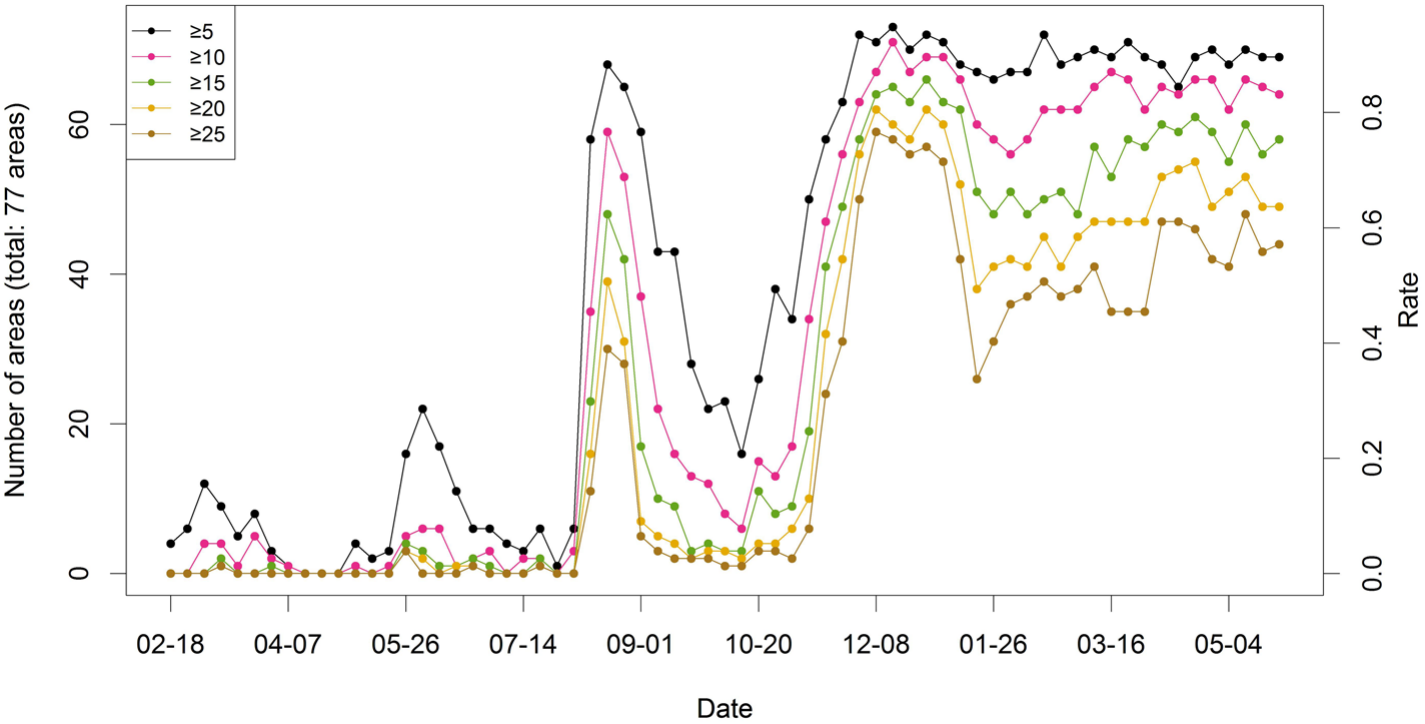
Time series plot for the number of metropolitan areas with the number of COVID-19 cases over a threshold in South Korea from February 18, 2020, to May 11, 2021.

The population in metropolitan areas in South Korea was approximately 25,674,800 as of 2018, making up more than 50% of the total population. The number of cases in metropolitan areas has been dominant since April 2020. Before cluster detection, we calculated the global Moran’s $I$ statistic for each week to examine the spatial spread in metropolitan areas (Fig. 7). There was statistical significance in many periods, such as August 2020 and May 2021. In addition, we counted the number of metropolitan areas with the number of cases over a threshold (Fig. 8). In August 2020, the number of areas with more than five cases dramatically increased to over 80% of the entire area. The rate has not dropped to less than 80% since December 2020. This implies that spatial spread occurred in metropolitan areas, supporting the need for a spatial investigation of the number of cases in metropolitan areas.

**Figure 9:**
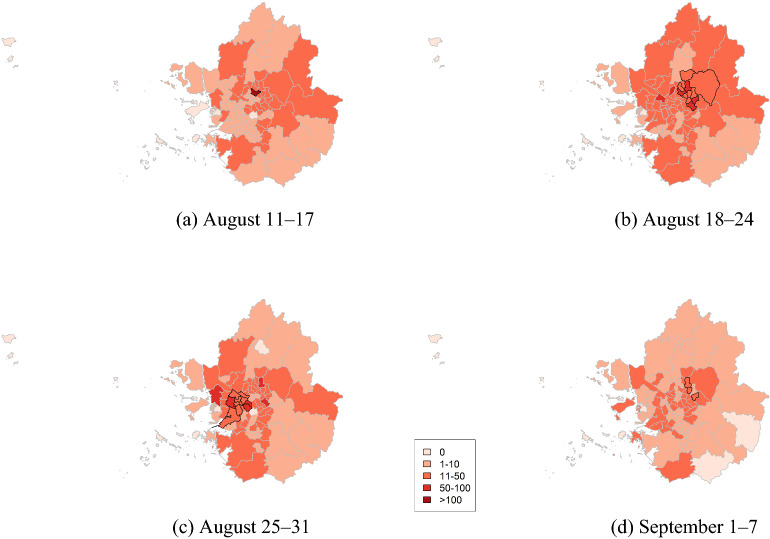
Cluster maps of COVID-19 cases for metropolitan areas in South Korea from August to September 2020.

We detected spatial clusters with elevated risks using a scan statistic for metropolitan areas from August to September 2020 (Fig. 9, Table 4). Most of the districts were in Seoul, and only some were in Gyeonggi.

**Table 4:** Cluster information of COVID-19 for metropolitan areas in South Korea from August to September 2020.

Week	Areas	Observed cases	Expected cases	Relative risk	p value
August 11–17	Seoul Seongbuk-gu	115	13.6	8.46	0.001
August 18–24	Seoul Seongbuk-gu and 7 other areas Gyeonggi Namyangju-si and 1 other area	470	216.49	1.83	0.001
August 25–31	Seoul Gangseo-gu and 6 other areas Gyeonggi Bucheon-si and 2 other areas	391	199.22	1.88	0.001
September 1–7	Seoul Gangdong-gu Seoul Jungnang-gu Seoul Nowon-gu	78	30.7	1.79	0.001

**Figure 10:**
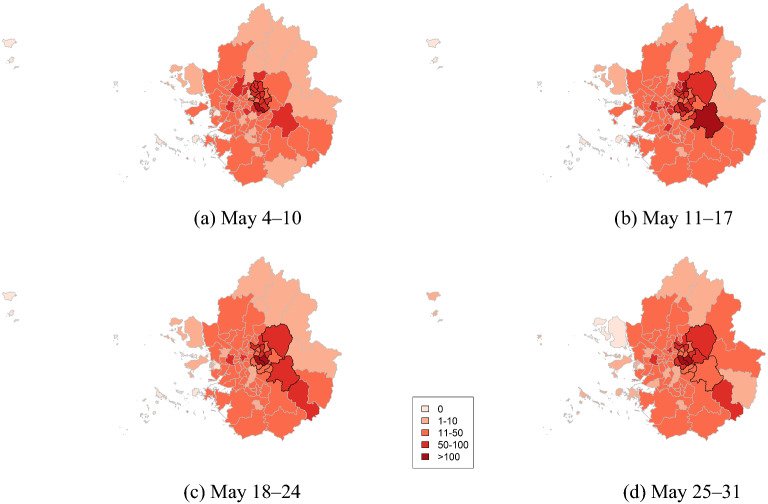
Cluster maps of COVID-19 cases for metropolitan areas in South Korea in May 2021.

The cluster sizes detected in May 2021 were larger than those detected in August–September 2020, and the number of cases in the detected clusters increased accordingly (Fig. 10, Table 5).

**Table 5:** Cluster information of COVID-19 for metropolitan areas in South Korea in May 2021.

Week	Areas	Observed cases	Expected cases	Relative risk	p value
May 4–10	Seoul Gangnam-gu and 11 other areas Gyeonggi Guri-si	824	420.05	1.84	0.001
May 11–17	Seoul Gangnam-gu and 8 other areas Gyeonggi Guri-si and 5 other areas	1015	547.01	1.86	0.001
May 18–24	Seoul Gangnam-gu and 8 other areas Gyeonggi Guri-si and 5 other areas	953	484.48	1.97	0.001
May 25–31	Seoul Gangnam-gu and 8 other areas Gyeonggi Gwangju-si and 4 other areas	872	450.18	1.84	0.001

## Discussion

In this study, we conducted a spatiotemporal analysis to investigate the spatial spread and time-varying clusters of COVID-19 in South Korea. Along with Moran’s I results, we presented various time series plots to examine the temporal pattern and produced choropleth maps to visually check the spatial association. To explore spatial clusters, scan statistics and visualization methods were considered. In general, the p value is related to sample size and significance^37^. It is possible to obtain small p values in large datasets with weak associations or large p values in small datasets with strong associations. Thus, we considered various visualization methods as well as statistical tests to investigate the spatial dynamics of COVID-19.

We found the areas in Daegu to be clusters in the early stage. This result may be due to mass infection in the Shincheonji religious group^38,39^. Then, metropolitan areas were detected as hotspots in December. It was reported that various cluster infections occurred in long-term hospitals, public saunas, and prisons in December 2020^40^.

Previous studies on the dynamics of the spatial patterns of COVID-19 have focused on existing spatial dependent effects or detecting spatial clusters, mainly using Moran’s I statistics and spatial scan statistics^3–5,25–27^. The spatial spread of COVID-19 in China at the very early stage, from January 16, 2020, to February 06, 2020, was first examined, using 31 province-level COVID-19 confirmed data^3^. The spatial patterns of COVID-19 in China from January 10, 2020, to March 5, 2020, was also studied^4^. The dynamic spatial association of COVID-19 in 31 province-level regions and 337 prefecture-level cities in China from January to October 2020 was examined^5^. In Iran, the spatial association and spatial hotspots of COVID-19 at the early stage (March and April 2020) was examined^25^, and the spatiotemporal patterns of COVID-19 from February 18 to October 21, 2020 were analyzed^26^. Approximately 4 months of the COVID-19 epidemic from January 20 to May 31, 2020, in South Korea were covered^27^. These studies mapped the spatial pattern and linked the clusters in the early epidemics, and the results may have contributed to knowledge on COVID-19 epidemics, especially during the period in which information about the virus was lacking. Our study included a longer period of 16 months and recent dates with more cases, so that it is a powerful approach for demonstrating the current dynamics of spatial clustering across South Korea.

The spatiotemporal dataset may contain excess zero counts owing to the spatiotemporal units; then, such property should be considered in the analysis. Here, we accounted for the excess zero counts by utilizing a zero-inflated Poisson distribution in the scan statistic. We used various spatiotemporal methods simultaneously, leading to better results than using only one method. We compared the results of different approaches and provided more comprehensive results. In this study, we conducted weekly spatial analysis to investigate the real-time spatial dynamics of COVID-19 cases across South Korea. Thus, we did not consider the use of multiple tests with p value adjustments^41^.

Despite the many strengths of this study, it has some limitations. First, we did not investigate possible confounding factors on COVID-19 spread. For example, the Korean government has implemented many social distancing policies and regulations. If we consider these nonpharmaceutical effects, we might obtain more precise results. In addition, we did not investigate the spatial association between COVID-19 and confounding factors, such as air pollution, weather, population mobility, and demographic characteristics. Thus, future research should investigate the effects of confounding factors on COVID-19 at the regional level in South Korea using statistical models.

Second, we used the official number of COVID-19 cases to study the spatial dynamics of COVID-19 in South Korea. However, the official numbers might be underestimated due to limited testing capacities, unexpected false negatives, overcrowding of hospitals, and unprepared health systems^42–48^. The spatial dynamics of COVID-19 using official numbers or real numbers might be different. Thus, it may be of interest to conduct spatiotemporal analysis of COVID-19 by considering the underestimation of COVID-19 cases.

## Conclusion

To the best of our knowledge, this is the first study to conduct a spatiotemporal analysis using long-term COVID-19 data in South Korea. Here, we showed that spatial spread of the coronavirus occurred, especially in metropolitan areas. A timely spatiotemporal analysis would be helpful for identifying hotspots and preventing spatial transmission of the virus during the pandemic.

## Data Availability

The data that support the findings of this study are available from the Korean Disease Control and Prevention Agency, but restrictions apply to the availability of these data, which were used under collaboration for the current study and are not publicly available. Data are however available from the authors upon reasonable request and with permission of the Korean Disease Control and Prevention Agency.

## Abbreviations

COVID-19: Coronavirus disease 2019
WHO: World Health Organization
Min: Minimum
SD: Standard deviation
Q1: First quartile
Q2: Second quartile
Q3: Third quartile
Max: Maximum

## References

[1] Riou J, Althaus CL (2020). Pattern of early human-to-human transmission of Wuhan 2019 novel coronavirus (2019-nCoV), December 2019 to January 2020. Eurosurveillance.

[2] World Health Organization. Coronavirus disease 2019 (COVID-19): situation report, 45. (2020). https://www.who.int/docs/default-source/coronaviruse/situation-reports/20200305-sitrep-45-covid-19.pdf. (accessed 14 Sep 2021).

[3] Kang D, Choi H, Kim JH, Choi J (2020). Spatial epidemic dynamics of the COVID-19 outbreak in China. Int. J. Infect. Dis..

[4] Li H (2020). Spatial statistical analysis of coronavirus disease 2019 (covid-19) in China. Geospat. Health.

[5] Wang Q (2021). Temporal and spatial analysis of COVID-19 transmission in China and its influencing factors. Int. J. Infect. Dis..

[6] James N, Menzies M, Bondell H (2021). Understanding spatial propagation using metric geometry with application to the spread of COVID-19 in the United States. Europhys. Lett..

[7] Desjardins MR, Hohl A, Delmelle EM (2020). Rapid surveillance of COVID-19 in the United States using a prospective space-time scan statistic: Detecting and evaluating emerging clusters. Appl. Geogr..

[8] Cuadros DF, Branscum AJ, Mukandavire Z, Miller FD, MacKinnon N (2021). Dynamics of the COVID-19 epidemic in urban and rural areas in the United States. Ann. Epidemiol..

[9] Wang Y, Liu Y, Struthers J, Lian M (2021). Spatiotemporal characteristics of the COVID-19 epidemic in the United States. Clin. Infect. Dis..

[10] Fronterre C (2020). COVID-19 in England: Spatial patterns and regional outbreaks. medRxiv..

[11] Elson R (2021). The spatio-temporal distribution of COVID-19 infection in England between January and June 2020. Epidemiol. Infect..

[12] Castro MC (2021). Spatiotemporal pattern of COVID-19 spread in Brazil. Science.

[13] Giuliani D, Dickson MM, Espa G, Santi F (2020). Modelling and predicting the spatio-temporal spread of COVID-19 in Italy. BMC Infect. Dis..

[14] Pluchino A (2021). A novel methodology for epidemic risk assessment of COVID-19 outbreak. Sci. Rep..

[15] Haug N (2020). Ranking the effectiveness of worldwide COVID-19 government interventions. Nat. Hum. Behav..

[16] Soucy JPR (2020). Estimating effects of physical distancing on the COVID-19 pandemic using an urban mobility index. medRxiv..

[17] Toharudin T (2021). National vaccination and local intervention impacts on COVID-19 cases. Sustainability.

[18] Chen S (2021). Climate and the spread of COVID-19. Sci. Rep..

[19] Christophi CA (2021). Ambient temperature and subsequent COVID-19 mortality in the OECD countries and individual United States. Sci. Rep..

[20] Fontal A, Bouma MJ, San-José A, López L, Pascual M, Rodó X (2021). Climatic signatures in the different COVID-19 pandemic waves across both hemispheres. Nat. Comput. Sci..

[21] Ganegoda NC, Wijaya KP, Amadi M, Erandi KKW, Aldila D (2021). Interrelationship between daily COVID-19 cases and average temperature as well as relative humidity in Germany. Sci. Rep..

[22] Ganslmeier M, Furceri D, Ostry JD (2021). The impact of weather on COVID-19 pandemic. Sci. Rep..

[23] Pegoraro V, Heiman F, Levante A, Urbinati D, Peduto I (2021). An Italian individual-level data study investigating on the association between air pollution exposure and Covid-19 severity in primary-care setting. BMC Public Health.

[24] Pan Y (2020). Quantifying human mobility behaviour changes during the COVID-19 outbreak in the United States. Sci. Rep..

[25] Shariati M, Mesgari T, Kasraee M, Jahangiri-Rad M (2020). Spatiotemporal analysis and hotspots detection of COVID-19 using geographic information system (March and April, 2020). J. Environ. Health Sci. Eng..

[26] Hazbavi Z, Mostfazadeh R, Alaei N, Azizi E (2021). Spatial and temporal analysis of the COVID-19 incidence pattern in Iran. Environ. Sci. Pollut. Res..

[27] Kim S, Castro MC (2020). Spatiotemporal pattern of COVID-19 and government response in South Korea (as of May 31, 2020). Int. J. Infect. Dis..

[28] Moran PA (1950). Notes on continuous stochastic phenomena. Biometrika.

[29] Jackson MC, Huang L, Xie Q, Tiwari RC (2010). A modified version of Moran's I. Int. J. Health Geogr..

[30] Oden N (1995). Adjusting Moran's I for population density. Stat. Med..

[31] Waldhor T (1996). The spatial autocorrelation coefficient Moran's I under heteroscedasticity. Stat. Med..

[32] Kulldorff M (1997). A spatial scan statistic. Commun. Stat. Theory Methods.

[33] Huang L, Kulldorff M, Gregorio D (2007). A spatial scan statistic for survival data. Biometrics.

[34] Kim AY, Wakefield J (2010). R Data and Methods for Spatial Epidemiology: The SpatialEpi Package.

[35] Allévius B (2018). Scanstatistics: Space-time anomaly detection using scan statistics. J. Open Source Softw..

[36] Paradis, E. *et al.* Package ‘ape’. *Analyses of Phylogenetics and Evolution. Version 2*. Retrieved from. https://cran.r-project.org/web/packages/ape/index.html (2019).

[37] Kühberger A, Lermer FA, Scherndl T (2015). The significance fallacy in inferential statistics. BMC Res. Notes.

[38] Korean Society of Infectious Diseases (2020). Report on the Epidemiological Features of Coronavirus Disease 2019 (COVID-19) outbreak in the Republic of Korea from January 19 to March 2, 2020. J. Korean Med. Sci..

[39] Lee J (2020). Epidemiological and clinical characteristics of coronavirus disease 2019 in Daegu, South Korea. Int. J. Infect. Dis..

[40] Jang JH (2021). Coronavirus disease-19 (COVID-19) one-year outbreak major cluster infection report as of January 19, 2021, in the Republic of Korea. Public Health Wkly. Rep.

[41] Greenland S, Hofman A (2019). Multiple comparisons controversies are about context and costs, not frequentism versus Bayesianism. Eur. J. Epidemiol..

[42] Baggiani A (2020). Management of healthcare areas for the prevention of COVID-19 emergency in an Italian teaching hospital in Pisa, Tuscany: A hospital renovation plan. Infect. Control Hosp. Epidemiol..

[43] Dinnes J (2021). Rapid, point-of-care antigen and molecular-based tests for diagnosis of SARS-CoV-2 infection. Cochrane Database Syst. Rev..

[44] Itamura K, Wu A, Illing E, Ting J, Higgins T (2021). Youtube videos demonstrating the nasopharyngeal swab technique for SARS-CoV-2 specimen collection: Content analysis. JMIR Public Health Surveill..

[45] Maniscalco, P. *et al.* The deep impact of novel CoVID-19 infection in an Orthopedics and Traumatology Department: The experience of the Piacenza Hospital. *Acta Bio-medica Atenei Parmensis* **91**, 97–105. 10.23750/abm.v91i2.9635 (2020). 10.23750/abm.v91i2.9635 PMC7569660 32420933

[46] Modi C, Böhm V, Ferraro S, Stein G, Seljak U (2021). Estimating COVID-19 mortality in Italy early in the COVID-19 pandemic. Nat. Commun..

[47] Richterich P (2020). Severe underestimation of COVID-19 case numbers: Effect of epidemic growth rate and test restrictions. medRxiv..

[48] Torres I, Sippy R, Sacoto F (2021). Assessing critical gaps in COVID-19 testing capacity: The case of delayed results in Ecuador. BMC Public Health.

